# Spatiotemporal heterogeneity in malaria transmission across Indonesia: analysis of routine surveillance data 2010–2019

**DOI:** 10.1186/s12916-025-03902-9

**Published:** 2025-03-05

**Authors:** Bimandra A. Djaafara, Ellie Sherrard-Smith, Thomas S. Churcher, Sri Budi Fajariyani, Hellen Dewi Prameswari, Herdiana Herdiana, Riskha Tiara Puspadewi, Karina D. Lestari, Iqbal R. F. Elyazar, Patrick G. T. Walker

**Affiliations:** 1 MRC Centre for Global Infectious Disease Analysis, Imperial College London, London, UK; 2 Oxford University Clinical Research Unit Indonesia, Faculty of Medicine, Universitas Indonesia, Jakarta, Indonesia; 3 Saw Swee Hock School of Public Health, National University of Singapore, Singapore, Singapore; 4 Department of Vector Biology, Liverpool School of Tropical Medicine, Liverpool, UK; 5 Malaria Working Group, Ministry of Health, Jakarta, Indonesia; 6 World Health Organization, Country Office for Indonesia, Jakarta, Indonesia

**Keywords:** Malaria, Indonesia, Spatiotemporal, Elimination, Surveillance

## Abstract

**Background:**

Indonesia faces challenges in achieving its goal of eliminating malaria by 2030, with cases stagnating between 2015 and 2019. This study analysed regional epidemiological trends and demographic changes in malaria cases from 2010 to 2019, considering differences in surveillance across the country.

**Methods:**

We analysed national and sub-national malaria routine surveillance data using generalised additive and generalised linear models to assess temporal trends in case reporting, test positivity, demographics, and parasite species distribution while accounting for surveillance variations.

**Results:**

After adjusting for increased testing from 2015 onwards, we estimated declining malaria incidence in six of seven Indonesian regions. These regions showed a demographic shift toward older, predominantly male cases, suggesting a transition from household to occupational transmission. In contrast, Papua maintained high transmission with cases concentrated in children. Despite comprising only 2% of Indonesia’s population, Papua’s contribution to national malaria cases rose from 40 to 90% (2010–2019).

**Conclusion:**

While most Indonesian regions progress toward elimination by addressing mobile and migrant populations and *P. vivax* transmission, Papua shows different patterns with persistently high transmission among children. Achieving nationwide elimination requires enhanced control measures, improved healthcare access, and strengthened multisectoral collaboration to address these region-specific challenges.

**Supplementary Information:**

The online version contains supplementary material available at 10.1186/s12916-025-03902-9.

## Background

Designing optimal malaria control strategies in Indonesia is a substantial challenge due to a multitude of complex epidemiological factors [1]. The diverse landscape of the country in terms of endemicity, population densities that range from dense urban areas to sparsely populated rural regions, a variety of malaria vectors with differing behaviours and bionomics, and the co-endemic presence of two dominant malaria species, *Plasmodium falciparum* and *Plasmodium vivax*, all contribute to this complexity [1, 2]. Additionally, concerns around zoonotic malaria parasite (*Plasmodium knowlesi*) infections [3] and the challenges of controlling malaria in mobile and migrant populations, particularly in areas nearing elimination [4], further complicate the situation.

Despite these challenges, from 2010 to 2019, Indonesia’s malaria elimination efforts made substantial progress. In 2017, more than half of the districts in the country—accounting for roughly 72% of the population—reported no local malaria transmission for three consecutive years, marking them malaria-free [5]. This achievement is largely attributed to the intensification of control efforts from the early 2000s and a National Ministerial Decree on Malaria Elimination in 2009 [6], which granted local authorities the autonomy and political backing to implement effective, locally tailored malaria control. The decree also precipitated improvements in aspects such as financing, the scaling up of artemisinin combination therapy (ACT), mass distributions of long-lasting pyrethroid-insecticide treated nets (LLINs), mandated laboratory confirmations, quality assurance for diagnoses, screening and treatment for pregnant women, and enhanced surveillance and reporting [5, 7].

After roughly ten years following the decree, it is crucial to objectively and quantitatively measure the impact of these endeavours in order to ensure the effective and efficient deployment of future control and elimination efforts. Routine surveillance data for malaria have increasingly been utilised to set national and regional targets, estimate disease burden, and measure the impact of control strategies [8]. In Indonesia, malaria case surveillance and reporting coverage improved significantly from covering only 26–50% of districts in 2010 to over 75% in 2015 [5]. However, these achievements in surveillance strengthening provide a challenge to interpreting true temporal patterns of underlying case trends within the reported data.

In this study, we leveraged a decade of routine malaria surveillance data from the Indonesia National Malaria Control Program (NMCP) to better understand the progress in malaria control and elimination efforts across the diverse Indonesian landscape, developing an inferential framework to adjust reported trends for the changes in surveillance capacity that occurred during the period. We characterise the overall trends in metrics such as case counts and test positivity ratios (TPR) and examine the shifts in malaria case profiles, including proportions by parasite species, and the age and sex of cases. We then compared their patterns to those that have been observed in other settings as they move toward malaria elimination, such as *P. vivax* becoming the dominant malaria species [9–11], an increase in the average age of clinical disease [12, 13], and an increase in the proportion of cases in men, as exposure becomes more occupational-driven [9, 14]. Through this multi-faceted lens, we aimed to develop a more comprehensive understanding of malaria transmission dynamics in Indonesia, providing detailed insights that can help explain the apparent plateau in national progress and inform targeted strategies needed to overcome persistent challenges in different regions.

## Methods

### Indonesia routine malaria surveillance data

We utilised monthly aggregated district-level malaria routine surveillance data from the SISMAL (*Sistem Informasi Surveilans Malaria* or Malaria Surveillance and Information System) platform of Indonesia NMCP from 2010 to 2019 [15]. For this study, monthly aggregated malaria cases and tests data were used. The total number of malaria diagnostic tests data is a combination of tests performed using either microscopy or rapid diagnostic test (RDT). The aggregated case data were grouped by age, sex, and parasite species. The age groups comprise 0–4 years old, 5–9 years old, 10–14 years old, and ≥ 15 years old. We classify the ≥ 15 years age group as ‘adults’ throughout this paper, as this age group in Indonesia, particularly in rural and endemic areas, typically engages in activities that influence malaria exposure patterns, including agricultural work and other occupational activities. While this classification includes some adolescents (15–17 years), it reflects the age at which individuals commonly begin participating in these exposure-relevant activities. The parasite species recorded were *P. falciparum*, *P. vivax*, *P. knowlesi*, *Plasmodium malariae*, *Plasmodium ovale*, and mixed infections of *P. falciparum* and *P. vivax*. However, the aggregated data did not cross-tabulate malaria cases across age groups or sex with parasite species.

### Estimating trends of malaria metrics from routine surveillance data

We used generalised additive models (GAMs) to produce estimates of overall regional trends adjusted for reporting at the district level throughout 2010–2019, while capturing complex non-linear trends between covariates and response variables using smooth functions or splines [16, 17]. Each region of Indonesia (Sumatra, Java and Bali, Kalimantan, Nusa Tenggara, Sulawesi, Maluku, and Papua) had a model fitted independently for each malaria metric, incorporating district and month-year as random effects covariate and a penalised smoothing spline covariate, respectively.

We employed GAM with a negative binomial family, to account for likely overdispersion in the distributions of cases and tests, and population counts as the model offset variable to model malaria cases and tests per 1000 population. Metrics and case profiles measured as proportions were modelled using GAMs with a binomial family and logistic link function. The mgcv package in R [18] was used for the implementation of GAM.

The malaria surveillance metrics and case profiles modelled are shown in Table 1, alongside their respective distribution families. To estimate the regional-level trend lines for all metrics, we calculated the weighted average values of all district-level trend lines within a region. The weighting factors used were (1) district-level population counts for modelled cases and tests; (2) modelled district-level tests for TPR; and (3) a combination of modelled district-level TPR multiplied by modelled tests for proportions of *P. vivax* cases, cases in males, and cases in ≥ 15 years old.

**Table 1:** The malaria surveillance metrics and cases profiles modelled and their respective distribution families

Malaria surveillance metrics and case profiles	Distribution family
Cases per 1000 population (overall and by age groups)	Negative binomial
Tests per 1000 population	Negative binomial
Test positivity ratio (TPR, %)	Binomial (logit)
Proportion of ≥ 15 years old cases (%)	Binomial (logit)
Proportion of cases in males (%)	Binomial (logit)
Proportion of *P. vivax* cases (%)	Binomial (logit)

### Modelling the relationship between age of malaria cases and malaria endemicity

The previous section measured the relationship between the age of malaria cases (i.e., the proportion of cases in adults) and malaria endemicity indirectly by comparing trends of both metrics over time. Here, we developed a framework using a generalised linear model (GLM) to directly analyse the relationship between malaria endemicity and age to see whether consistent patterns were observed across all regions of Indonesia. We assumed a model whereby the Annual Parasite Incidence (API) per 1000 (at the log-scale) alters the mean age of reported malaria cases (*μ*) on the geometric scale. The GLM was fitted to the proportion of malaria cases by age groups (0–4, 5–9, 10–14, and ≥ 15 years old). Each year’s district-level malaria case data were used for the model fitting process, filtering only observations with at least 30 malaria cases reported to reduce the noise from the low-level malaria case counts.

## Results

**Fig. 1:**
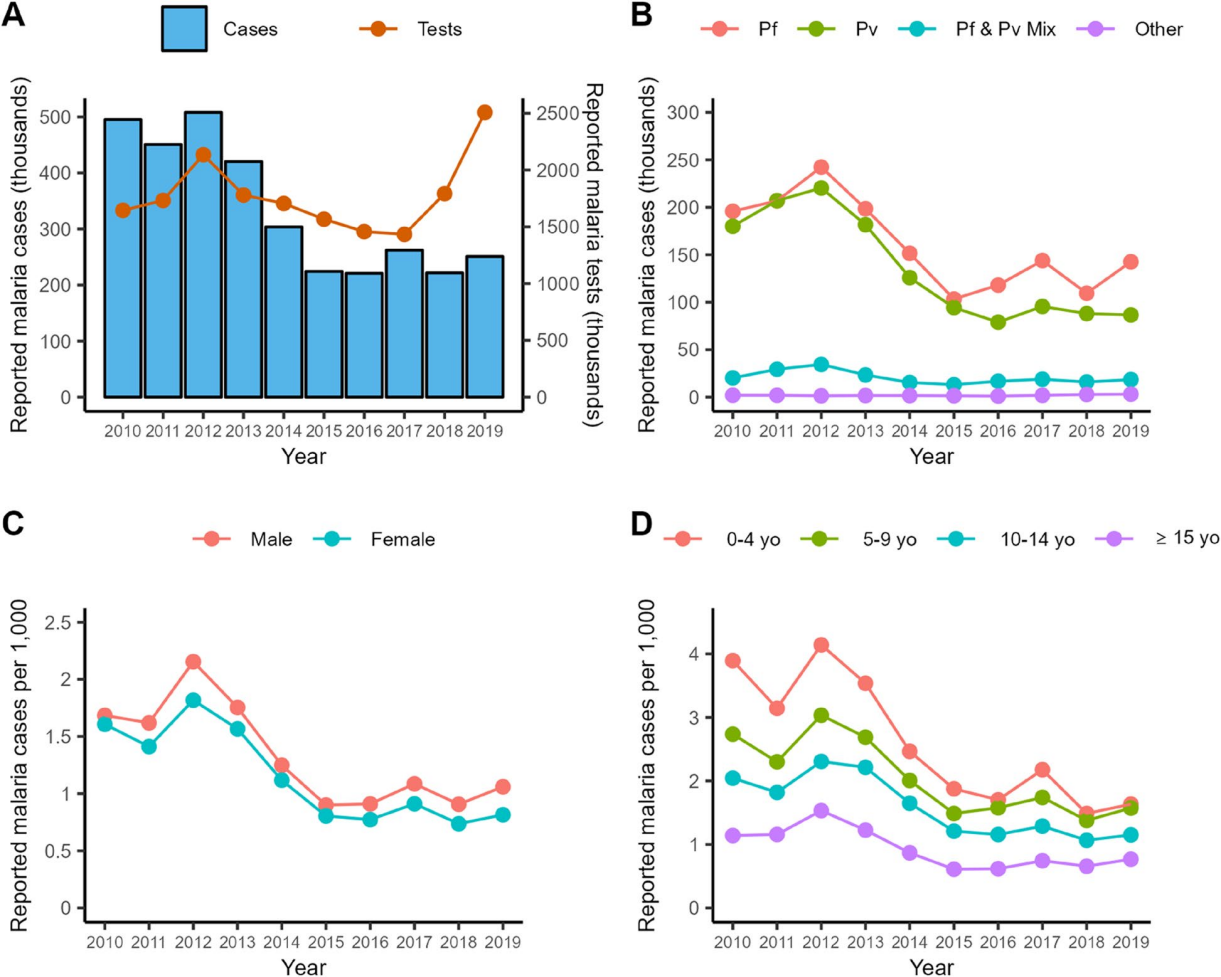
National-level trends of several malaria metrics, calculated yearly, derived from routine malaria surveillance data. **A** reported malaria cases and tests performed; **B** reported malaria cases by parasite species; **C** reported malaria cases by sex; and **D** reported malaria cases by age groups

Figure 1 shows the national-level trends of several malaria metrics derived from the routine malaria surveillance data between 2010 and 2019. The number of reported malaria cases was reduced by half during this period, while malaria tests, which reflect surveillance efforts, almost doubled within the same period (Fig. 1A). Nationwide, *P. falciparum* and *P. vivax* are the dominant malaria parasite species, with *P. falciparum* reported more frequently throughout the decade (Fig. 1B). Malaria cases per capita were found more frequently in males (Fig. 1C) and the youngest age groups, 0–4 and 5–9 years old (Fig. 1D). However, those in the youngest age groups also experienced the largest declines in the per capita malaria rate over the decade.

**Fig. 2:**
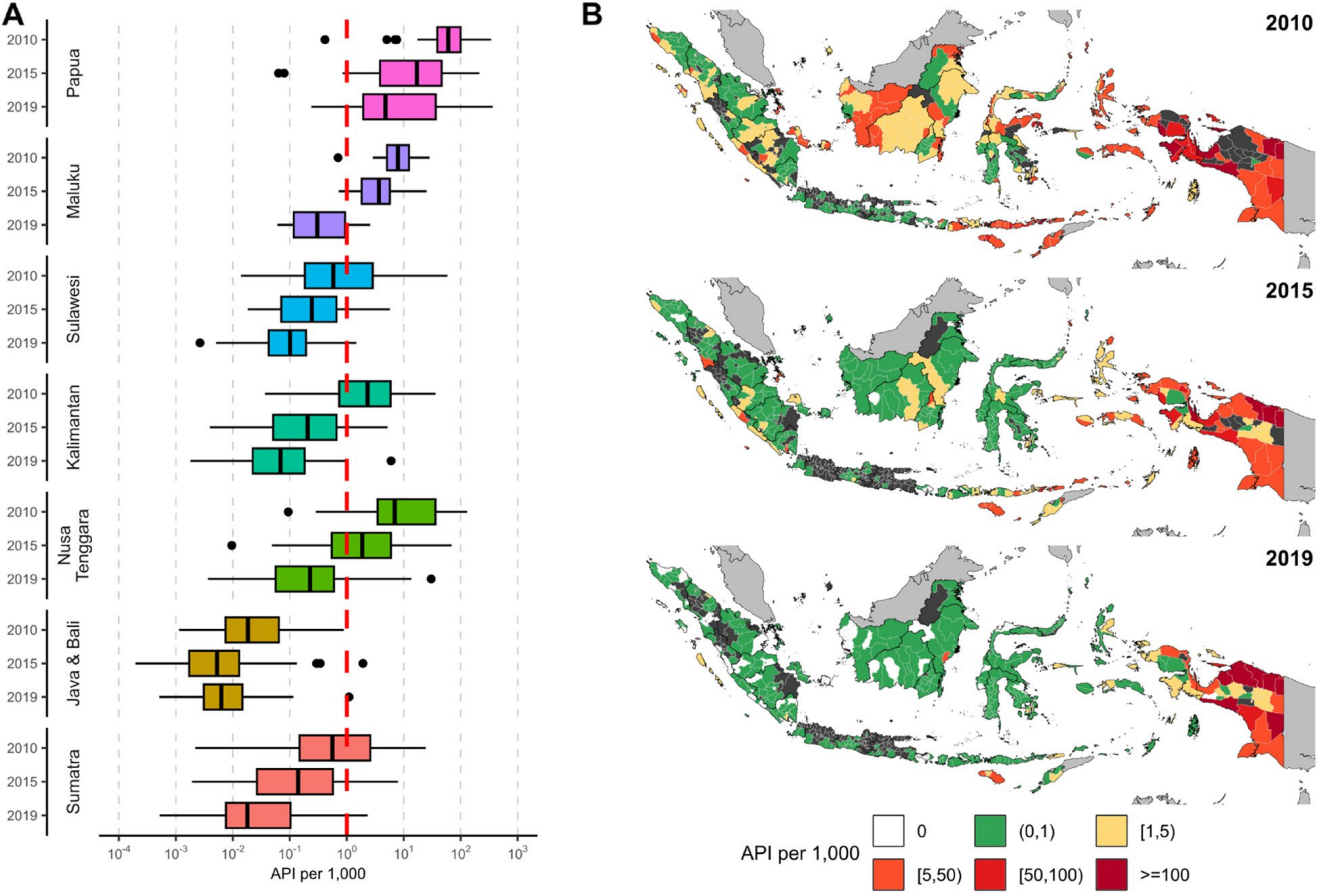
Distributions of malaria incidence in Indonesia. **A** Within-region boxplots of API per 1000 in 2010, 2015, and 2019; and** B** geographical distributions of API per 1000 at the district level in 2010, 2015, and 2019. Dark greys denote no data was available

The geographical heterogeneity of malaria transmission in Indonesia is shown in Fig. 2. In most regions, both median and maximum district-level malaria endemicity and maximum district-level incidence fell steadily throughout the study period (Fig. 2A). However, some districts, particularly in the Papua region (the easternmost region), showed considerable differences in trends. Despite overall showing a progressive reduction in median district-level malaria endemicity, some districts reported similar or higher API per 1000 in 2019 relative to 2010 (Fig. 2B).

**Fig. 3:**
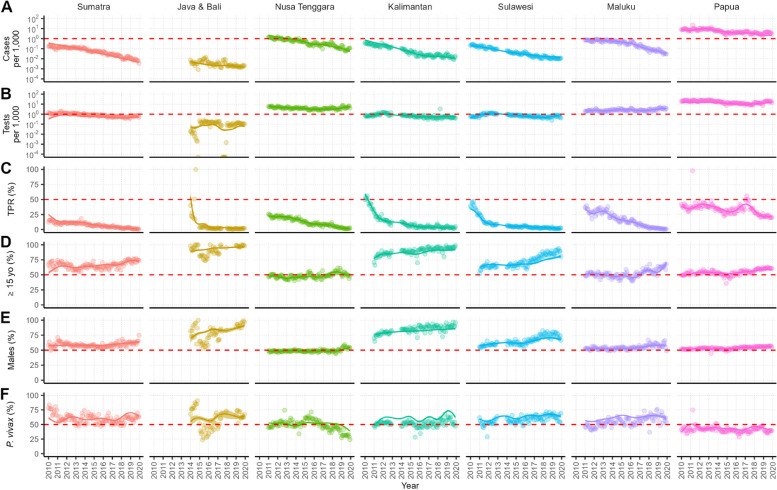
Regional-level monthly trends of several malaria metrics derived from routine malaria surveillance data. Solid lines and the shaded areas denote the median and 95% credible intervals of the modelled trends using GAM. The semi-transparent points denote region-level monthly averages from data. The metrics shown are **A** cases per 1000; **B** tests per 1000; **C** TPR (%); **D)** proportion of cases in ≥ 15 years old age group (%); **E** proportion of cases in males (%); and **F** proportion of *P. vivax* cases (%). Red dashed line is included as a fixed value to aid comparison between areas

Figure 3 shows the region-level trends of several malaria metrics estimated by GAM between 2010 and 2019, adjusting for reporting rates by district across the study period. Over the decade, adjusted malaria incidence rates declined in all regions of the country (Fig. 3A). The declining trends differ from one region to another in terms of their baselines and slopes. Hence, there are differences in the magnitudes of the decline, with the highest reduction magnitude estimated in the Sumatra region (81.5-fold reduction) and the lowest in the Papua region (3.6-fold). Cases per 1000 population trends for each age group are shown in Additional file 1: Fig. S1. These declines in malaria cases per 1000 population were estimated despite case-finding efforts remaining stable across all regions (Fig. 3B). The estimated trends of testing efforts are shown on a per 1000 population basis, meaning that, in absolute terms, as the population grew over the years, the testing efforts increased. The substantial reduction in malaria burden across all Indonesian regions is also supported by the estimated test positivity ratio (TPR) trends, which show declines across all regions (Fig. 3C). However, in the Papua region, we observed an increase in adjusted TPR in 2017 before declining again in the following years.

Cases were generally found in older populations, with increasing proportions of malaria cases in adults over the years (Fig. 3D). However, on a per capita basis, malaria burden in children is still the highest (Fig. 1D). Furthermore, malaria cases have become increasingly male-dominant (Fig. 3E), which could indicate a shift towards a higher proportion of occupational exposures. In terms of malaria parasite species, there is no sign that *P. vivax* became the largely dominant parasite species in any region (Fig. 3F), despite some regions experiencing slight shifts in species distribution. Notably, Nusa Tenggara is the only region where there has been a consistent decline in the proportion of *P. vivax* infections in the last years of the decade. Papua, on the other hand, is the only region with consistent *P. falciparum*-dominant infections in the country.

**Fig. 4:**
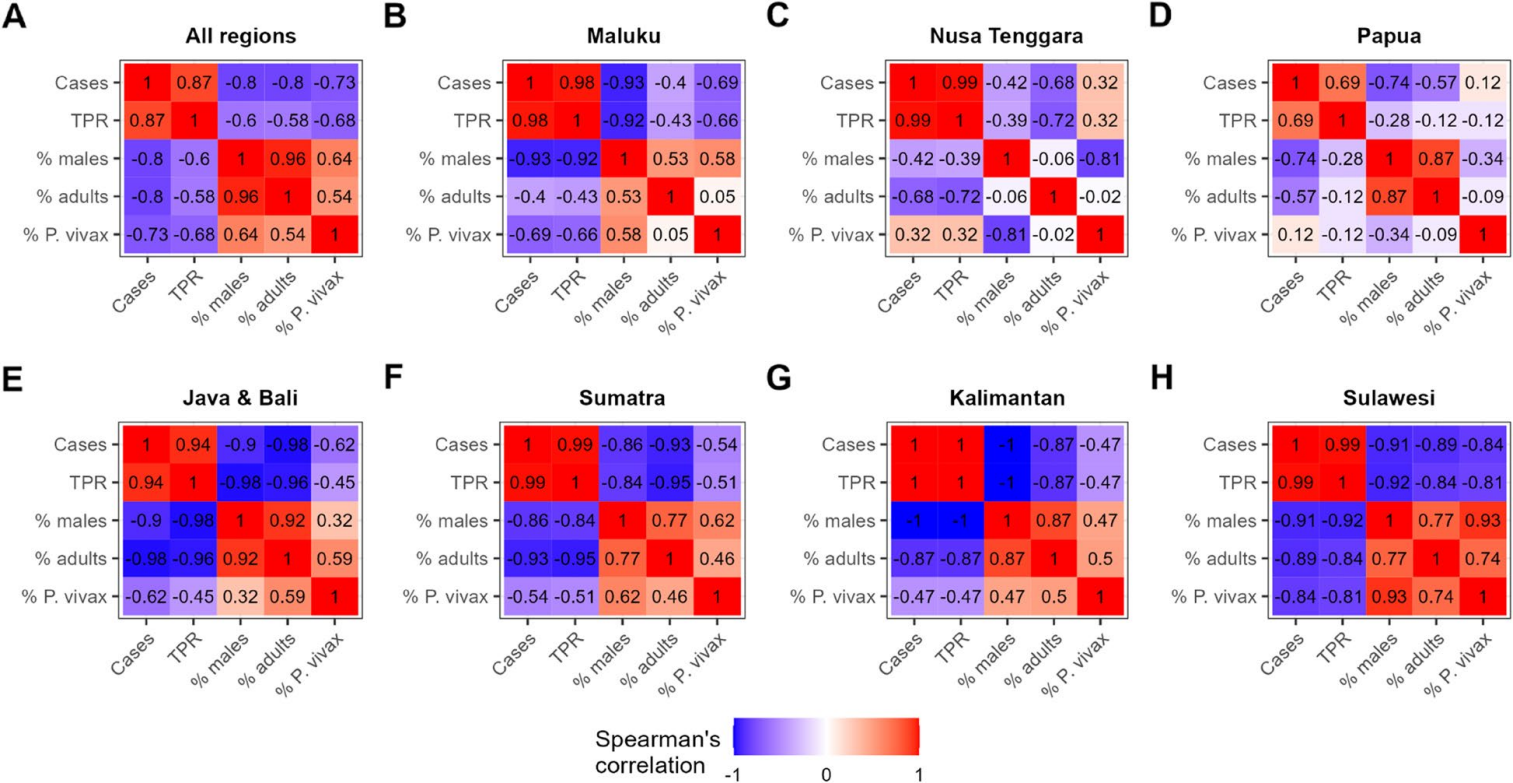
Spearman’s rank correlations between the modelled monthly estimates of malaria metrics using GAM. Red denotes positive correlations, while blue denotes negative correlations between metrics. **A** All regions; **B**, **C** and** D** regions with the highest malaria endemicity: Maluku, Nusa Tenggara, and Papua, respectively; **E**, **F**,** G** and** H** regions with the lowest malaria endemicity: Java and Bali, Sumatra, Kalimantan, and Sulawesi

The relationship between the different adjusted metric trends was estimated using Spearman’s rank correlation, combining model estimates from all regions and for each region (Fig. 4). There is a strong positive correlation between malaria cases and TPR at both national and regional levels, though somewhat less so in the Papua region. Meanwhile, decreases in both cases and TPR showed a correlation with increases in the proportion of cases that were adult, which itself was largely synchronised with increases in the proportion of cases that were male. This trend of cases becoming typically older and male as transmission declines was particularly strong in regions of historically lower endemicity (Java and Bali, Sumatra, Sulawesi, Kalimantan). National-level increases in the proportion of cases that were *P. falciparum* (Fig. 1B) mask high regional-level correlations between declines in transmission and the increasing role of *P. vivax* in all but the two regions with the highest burden (Nusa Tenggara and Papua).

**Fig. 5:**
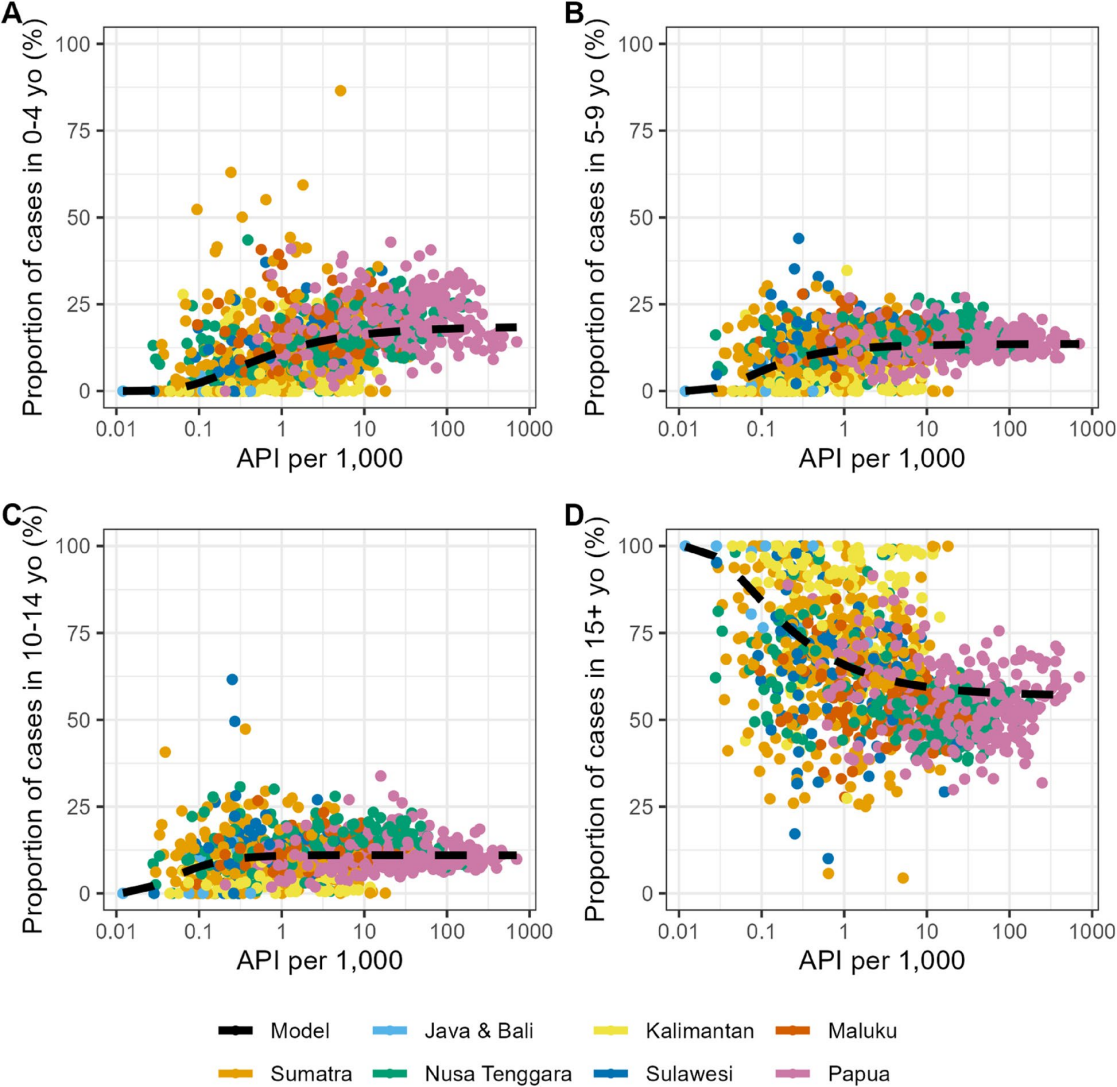
Modelled relationships between API per 1000 and the proportion of cases in **A** 0–4 years old; **B** 5–9 years old; **C** 10–14 years old; and **D** ≥ 15 years old, with overlaid data from routine surveillance. Dashed lines denote the median of generalised linear model (GLM) estimates of the relationship. The colours of the data points represent regions

We investigated how malaria endemicity (as measured by API per 1000) shapes the age-profile of reported cases using GLM. Figure 5 illustrates the relationship between API per 1000 and the proportion of cases in the population aged 0–4, 5–9, 10–14, and ≥ 15 years old. In low-endemic settings (for example, Java and Bali), cases are dominated by adults, but the proportion of cases in children increases as endemicity increases. Model parameters convergence and validation, as well as the proportion of cases by age group (in selected districts representing the upper, middle, and lower quantiles of API), are shown in Additional file 1: Figs. S2–S4. Additional file 1: Fig. S5 shows the combined modelled relationship between API per 1000 and the proportions of those age groups.

**Fig. 6:**
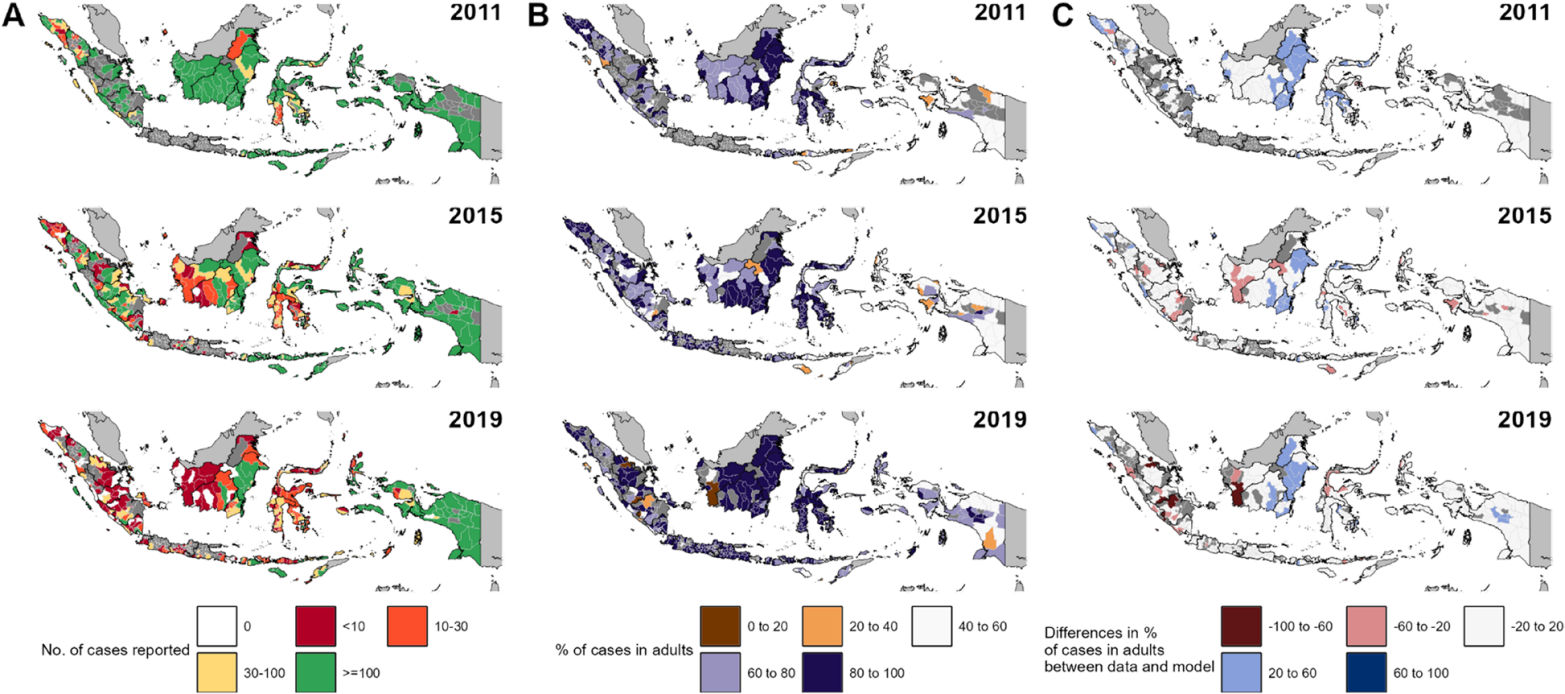
Maps highlighting the proportion of malaria cases in adults in Indonesia. The top, middle, and bottom rows represent 2011, 2015, and 2019, respectively. **A** District-level maps of the number of malaria cases reported within a year, serving as ‘sample size’ of the calculated proportions from data. Districts reporting low case counts are coloured in red, representing low sample sizes to infer proportions presented in **B**, while districts reporting high case counts are coloured in green, representing high sample sizes; **B** district-level maps of the reported proportion of malaria cases in adults. Brown colours denote districts where children dominated the reported malaria cases, while districts coloured in purple denote adult-dominant malaria cases; and **C** District-level maps of the difference between the proportion of malaria cases in adults based on reported data and the model average, as shown in Fig. 5. Red colours denote districts with lower proportions of cases in adults than the model average, while blue colours denote districts with proportions of cases in adults higher than the model average

Finally, Fig. 6 shows the geographic distribution of outliers to the GLM results, whereby model estimates of the proportion of adults, generated using district-level case counts (Fig. 6A) are compared to those observed in the data (Fig. 6B). As Fig. 6C shows, 84% of 400 districts reporting their malaria data in 2019 fall within the − 20% to 20% difference bracket between data and model estimates, which interval arbitrarily chosen to visualise ‘no difference’ between them. Those that lie beyond this threshold include clusters of districts within Sumatra in 2019 that coincide with some of the steepest declines in API in the study period, where a higher proportion of children than expected appear in case data than expected by the model. A similar pattern is also seen in low-endemic districts in western Kalimantan. This contrasts, however, eastern Kalimantan where districts often report higher than expected cases in adults throughout the study period.

## Discussion

Our analysis highlights the heterogeneity in progress towards malaria elimination across Indonesia despite a major decline in malaria cases that occurred nationally between 2010 and 2019. While national-level malaria data would suggest stagnation in progress since 2015, sub-national trends tell a different story. In regions covering more than 95% of the country’s population, malaria cases have steadily decreased. However, trends in raw national-level data have become increasingly dominated by high-endemic regions such as Nusa Tenggara, Maluku, and Papua, where only 7% of the population resides (19 million people) but which represented 95% of reported malaria cases in 2019, rising from 73% in 2010 (Papua region, 2% population, ~ 40% contribution to ~ 90%).

When data are considered at the region level, further divergence in trends between regions with lower and higher baseline endemicity emerges. In the four lowest endemicity regions (Java and Bali, Sumatra, Sulawesi, Kalimantan), we observed clear patterns of a steadily rising proportion of cases in males and adults as transmission has declined. These findings typically indicate occupational-driven exposure, where transmission occurs in high malaria-risk settings such as forests and mines, far from human settlements [4, 19, 20]. This suggests that control strategies in these regions may need to be reoriented to address these particular demographic groups more effectively.

Our analysis of the relationship between surveillance metrics revealed important patterns that could help identify districts where control measures may not work as expected. When considering the relationship between case incidence and the age of people with reported cases, we found distinct patterns of younger-than-expected age patterns emerging in clusters of districts in the northern province of Aceh in Sumatra and the western provinces of Kalimantan. Many of these districts had experienced some of the highest declines in malaria incidence over the past decade (i.e., from API per 1000 > 100 to API per 1000 < 10). This may implicate a role of residual immunity in adults [12], so this outlier status may prove transient. However, for some districts in the central region of Sumatra, deforestation due to increased mining and plantation activities has also increased malaria risks in the nomadic indigenous population, with malaria prevalence as high as 24% [21]. Such pockets of community-based transmission, in a wider landscape of largely occupational exposure, would also contribute to the younger-than-typical demographics of observed cases in the region. In contrast, the largest clusters of older-than-expected age distributions were found in districts with API > 10 in eastern Kalimantan, an area characterised by occupational-driven exposure through agriculture and forest-related activities [20, 22–24]. These contrasting age patterns highlight both the importance of protecting indigenous populations and addressing the ecological and economic factors that drive occupational exposure in different regions.

Treatment and drug resistance monitoring remain crucial elements of Indonesia’s elimination strategy. During the study period, Indonesia’s first-line antimalarial treatment consisted of dihydroartemisinin-piperaquine (DHA-PPQ) with primaquine (PQ), while non-ACT combinations (quinine with clindamycin/tetracycline and PQ) served as second-line treatment [25]. Following World Health Organization (WHO) recommendations, the Ministry of Health conducts regular therapeutic efficacy studies (TES) every 2 years in endemic areas [26–28]. While recent TES results have demonstrated that DHA-PPQ remains effective, continued vigilance through molecular surveillance is crucial given the spread of artemisinin partial resistance beyond Southeast Asia.

The increasing proportion of *P. vivax* cases supports the need for effective approaches to achieving radical cures and eliminating the hypnozoite reservoir. Indonesian guidelines recommend a low-dose regimen for PQ (3.5 mg/kg total dose; 0.25 mg/kg/day for 14 days) without universal G6PD testing for most cases [25], though low adherence remains a significant challenge. While single-dose tafenoquine (TQ) offers potential advantages for adherence, its co-administration with DHA-PPQ showed limited clinical benefit in one recent trial [29], reducing the risk of relapse compared to DHA-PPQ alone (21% vs 11%). It is also still significantly inferior to the current Indonesian regimen of PQ plus DHA-PPQ and notably lower than in previous studies of TQ with chloroquine (though resistance towards it has been observed in Indonesia [30]). Alternative approaches, including a shorter 7-day PQ regimen, might improve adherence compared to the standard 14-day course [31], but both TQ and shorter PQ regimens require G6PD testing at health facilities. To address this requirement, a feasibility study is currently planned in Indonesia for point-of-care testing using the STANDARD G6PD test [32]. For G6PD-deficient individuals, weekly PQ dosing presents a safer alternative and is recommended by the national guidelines [25, 33]. Community health centre-based strategies, such as directly observed therapy, have shown promise in improving treatment adherence [34, 35], though addressing structural barriers remains crucial for success [36].

The Papua region presents the greatest challenge to Indonesia’s elimination goals. Despite a decade of vector control scale-up, transmission remains firmly embedded within communities. Multiple barriers hinder progress, including poor quality and uneven provision of health services, lower socioeconomic status, and local political instability [37]. While some health indicators have improved, the region continues to lag behind western Indonesia. The situation varies at the local level, with districts in western Papua showing sustained reductions while districts in eastern Papua experienced resurgences in API. This resurgence, partly attributable to increased testing, also mirrors trends in neighbouring Papua New Guinea [38, 39], suggesting potential cross-border transmission challenges.

To address these challenges, novel interventions are being explored in Papua. Mass drug administration campaigns were conducted in 2023, and promising results have been seen with intermittent preventive treatment of malaria in pregnancy (IPTp) using DHA-PPQ, which reduced malaria in pregnancy by 77% compared to single screening and treatment (SST) approach in a clinical trial [40]. The Ministry of Health plans to expand IPTp with DHA-PPQ to districts with API > 50. Vector control remains crucial, with annual insecticide resistance monitoring showing that first-generation pyrethroid-based LLINs remain effective as resistance is still sporadic [41]. Building on the comprehensive vector mapping from the RIKHUS VEKTORA program (2015–2017), which characterised vector distribution, behaviour, and habitat preferences across 90 districts, longitudinal vector surveillance efforts in Papua have been discontinued due to security concerns and funding constraints. While the expert committee has not recommended current malaria vaccines (RTS, S and R21) due to low severe disease burden and poor immunisation coverage, other interventions such as larval source management and future vaccine development targeting adults and *P. vivax* could strengthen control efforts.

Regional heterogeneity presents additional complexities in some areas. In Nusa Tenggara and Maluku, trends in endemicity levels, case demographics, and *Plasmodium* species composition show less clear relationships when aggregated regionally, largely due to high between-province and between-district heterogeneity. For instance, in Nusa Tenggara, the counter-intuitive increase in *P. vivax* proportion despite overall transmission decrease can be explained by cases becoming increasingly concentrated in the southern archipelagic islands of Sumba and Timor (86% of cases in 2019), where transmission decline has been slower and species composition remains stable. In contrast, the remainder of the region (75% of the population) shows sustained declines with increasing *P. vivax* contribution, matching trends in other low-transmission regions.

Understanding the drivers of this heterogeneous landscape is complex, particularly as malaria control scale-up has coincided with multiple ongoing environmental and societal changes. These include land-use changes from agricultural expansion and deforestation [42–44], and regional variations in climate factors such as temperature, rainfall, and humidity [45]. Additional challenges may emerge from the capital relocation project to East Kalimantan, which could increase local transmission risk due to the influx of malaria-naïve populations and proximity to endemic districts [46].

Surveillance of other Plasmodium species requires attention. In the database we analysed, we observed an increase in *P. malariae* and *P. ovale* proportions from 0.5% (2010) to 1.2% (2019), though it was unclear whether this represents increased transmission or improved surveillance. Similarly, 283 reported *P. knowlesi* cases (2010–2019) were much lower than the 545 cases identified in a recent review of publications which included studies utilising molecular testing [46]. Given that misdiagnosis is common in areas with a high risk of *P. knowlesi* infections [47, 48], and this species now dominates the neighbouring Malaysia Borneo [49], enhanced diagnostic and surveillance capacity is needed, potentially through additional microscopist training or strategic deployment of molecular testing [47, 50].

The path forward requires strengthened collaboration. The success of Indonesia’s malaria elimination efforts will depend on enhanced inter-district within-country and cross-border collaborations. Successful models exist in the Greater Mekong Subregion, countries have established joint malaria elimination initiatives that harmonise surveillance, share data, and align intervention strategies along shared borders [51]. Such cooperation has proven critical for managing malaria cases in mobile and migrant populations that can serve as malaria reservoirs. Adapting these approaches to the Indonesian context could help address the remaining challenges in achieving nationwide elimination.

## Conclusion

In conclusion, while Indonesia has made significant progress towards malaria elimination over the past decade, our analysis reveals distinct challenges across the country’s heterogenous malaria settings. In low transmission settings (Sumatra, Java and Bali, Kalimantan, and Sulawesi), the primary challenges involve managing mobile and migrant populations and addressing *P. vivax* as the dominant species. High transmission areas (Nusa Tenggara, Maluku, and Papua) require both innovative interventions and improvements in underlying socioeconomic conditions and healthcare access. Success in both contexts will require strengthened inter-district and cross-border collaborations to prevent malaria importation into areas approaching elimination. Additionally, enhanced diagnostic and surveillance capacity is crucial for monitoring potential zoonotic malaria transmission, particularly *P. knowlesi*, which has emerged as a significant concern in neighbouring countries.

This study also underscores the importance of understanding limitations in routine surveillance data when interpreting malaria trends. While current data limit the depth of insights due to a lack of granular information linking cases to demographic factors and infection sources, new opportunities are emerging. The electronic system implemented by the National Malaria Control Program in 2019 captures individual-level data, offering prospects for richer insights to guide elimination efforts, provided data quality and reporting challenges are addressed.

## Supplementary Information

Additional file 1. Detailed modelling methods and supplementary figures. Figure S1: Regional-level monthly trends of malaria cases per 1,000 by age group. Figure S2: Trace plots for GLM fitting in Stan for all fitted model parameters. Figure S3-S4: GLM fitting performance compared to data. Figure S5: Modelled relationship between API per 1,000 and proportion of malaria cases by age group.

## Data Availability

The surveillance data that support the findings of this study are available from the Malaria Working Group at the Ministry of Health of Indonesia. Requests for data access can be directed to the Malaria Working Group, Ministry of Health, Republic of Indonesia.

## Abbreviations

ACT: Artemisinin combination
API: Annual Parasite Incidence
DHA-PPQ: Dihydroartemisinin-piperaquine
GAM: Generalised additive model
GLM: Generalised linear model
G6PD: Glucose-6-phosphate dehydrogenase
IPTp: Intermittent preventive treatment of malaria in pregnancy
LLIN: Long-lasting pyrethroid-insecticide-treated net
NMCP: National Malaria Control Program
PQ: Primaquine
RDT: Rapid diagnostic test
RIKHUS VEKTORA: *Riset Khusus Vektor dan Reservoir Penyakit* Or Special Research on Vector and Reservoir of Diseases
SISMAL: *Sistem Informasi Surveilans Malaria* Or Malaria Surveillance and Information System
SST: Single screening and treatment
TES: Therapeutic efficacy study
TQ: Tafenoquine
TPR: Test positivity ratio
WHO: World Health Organization
